# Supplementary material for: Nudge strategies for behavior-based prevention and control of neglected tropical diseases: A scoping review and ethical assessment
Source: PLoS Negl Trop Dis. 2021 Nov 1;15(11):e0009239. doi: 10.1371/journal.pntd.0009239 (PMC8584752; doi:10.1371/journal.pntd.0009239)
Supplement: Attachment — Submitted filename: Review of PNTD-D-21-00223.docx [file pntd.0009239.s003.docx]

Review of manuscript (No. PNTD-D-21-00223)

Date: April 09, 2021

**Title**

Nudge strategies for behavior-based prevention and control of neglected tropical

Thank you for the opportunity to review this manuscript. I think this paper is most informative and relevant to research and techniques in developing countries. Reviews, systematic or in this case scoping reviews, are a crucial part of the literature serving to contextualize what is known and outlining the gaps in our knowledge.

The focus on NTDs made for an exciting topic area because, as the name implies, this group of diseases is "neglected." Though it may be worth discussing if some of the diseases classified as NTDs remain neglected.

Please see my comments below for some suggested changes or adjustments that may help improve the manuscript.

**Introduction**

- Authors ought to give examples of what they mean by NTDs, especially since in the first paragraph, they list several disease categories in addition to NTDs.
- Have other reviews covered this area; systematic, scoping, or even a general review? There may be no SRs for "nudge strategies for infectious diseases," but there may be SRs on nudge strategies or SRs on NTDs/infectious diseases that may yield some valuable information.

**Methods**

- This part of the document is well written, and I don't have anything to add. I thought the authors were accurate in the adherence to the PRISMA-SR framework, and it served them well.

**Discussion**

- I like that the authors put forward an ethical framework to aid in choosing nudge interventions. However, the section was difficult to follow along, and I encourage the authors to consider some kind of illustrative representation of their proposed framework. A tabular representation or some figure would be a practical addition to this manuscript. Furthermore, some kind of visual will be much easier to disseminate.
- For example, you can have a table with three columns, High, Moderate, and Low. Each row is a criterion in the ethical framework, and relevant definitions are in each cell.
- Given that the manuscript began with an argument about policy and agenda setting regarding NTDs, I think the discussion could benefit from a call back to the introduction. How might the findings contribute to further narrowing the policy and health agenda environments, either in specific countries, regions or globally?

**Figures**

- Add some figure legends and labels to the figures you have at the end of your manuscript to explain what they mean. Typically, figures should serve as stand-alone from the text in that your reader should understand what they mean with minimal references to the text.
- The systematic flow diagram is a bit confusing. A flow diagram should not have many arrows and boxes intersecting. Additionally, boxes in one category should not bleed over into others. For instance, in the Eligibility band, you have a box for Records Excluded after the full-text review (n=35). This 'Records Excluded' box extends into the 'Inclusion' band. Ideally, the only information in that band should be the' Studies included (n=33)' box.
- The flow diagram should also avoid using arrows with hatched lines that intersect with arrows with solid lines. For example, what are the arrows extending from the 'Records excluded (n=135)' back to the Full-text articles remaining box supposed to mean? If there are multiple reviews of the excluded text, then build those into the hierarchy vertically and lengthen the eligibility band.
- Check the below website for a PRISMA flow chart generator. I have found it helpful in the past. While this is a scoping review, the PRISMA flowchart typically used in systematic reviews can be effectively modified to suit this study's needs. <http://prisma.thetacollaborative.ca/>
