# Supplementary material for: Nudge strategies for behavior-based prevention and control of neglected tropical diseases: A scoping review and ethical assessment
Source: PLoS Negl Trop Dis. 2021 Nov 1;15(11):e0009239. doi: 10.1371/journal.pntd.0009239 (PMC8584752; doi:10.1371/journal.pntd.0009239)
Supplement: Attachment — Submitted filename: Cover letter_responses.docx [file pntd.0009239.s004.docx]

**Cover letter - responses to the reviewer comments to submission PNTD-D-21-00223**

**Title:** Nudge strategies for behavior-based prevention and control of neglected tropical diseases: a scoping review and ethical assessment

Dear Editors,

We are indebted and would like to extend our gratitude to the editors and reviewers for the positive feedback provided, as well as the thorough consideration and constructive comments on our manuscript. Below we have systematically addressed the comments made by the reviewers and describe how the suggestions and changes have been incorporated in the revised version of the manuscript. Sections in blue font represent our responses to each inquiry, changes in the manuscript are highlighted and indicated by line accordingly.

We look forward to receiving any additional feedback or comments on our revised manuscript. Please do not hesitate to get in touch if you have any further questions.

Yours sincerely,

Fiona Vande Velde

Postdoctoral fellow, Dept. of Public Health Sciences, Norwegian University of Life Sciences

**REVIEWER 1**

**Introduction**

Authors ought to give examples of what they mean by NTDs, especially since in the first paragraph, they list several disease categories in addition to NTDs.

As suggested by the reviewer, we included a statement (L94 – 97) of what constitutes an NTD in the first sentence, based on a recent paper published in PlosNTDs: Hotez PJ, Aksoy S, Brindley PJ, Kamhawi S (2020) What constitutes a neglected tropical disease? PLoS Negl Trop Dis 14(1): e0008001. Doi: 10.1371/journal.pntd.0008001

L94 – 97: “In recognition of the underinvestment, global attention and commitment to address neglected tropical diseases (NTDs), the United Nations formally recognized the NTDs, a diverse group of infectious diseases that are common in low-income populations with poor access to quality health services, water, sanitation and hygiene, generally being overlooked and receiving little attention [1].”

Have other reviews covered this area; systematic, scoping, or even a general review? There may be no SRs for "nudge strategies for infectious diseases," but there may be SRs on nudge strategies or SRs on NTDs/infectious diseases that may yield some valuable information.

Inputs were used from other reviews/review protocols on nudge strategies and on infectious diseases to guide the focus of this review as well as other methodological decisions. We have listed the sources of our choices underneath and state this more clearly in the manuscript

Definition of Hansen: O’Keeffe et al 2019 (L133 – 134)

Search strategy: Möllerkamp et al 2019 (L189 – 190)

Categorizing the nudges based on Müncher’s taxonomy: Forberger et al 2019 (L248 – 249)

Study selection: Weston et al. 2018 (L295)

In addition, we have added a section (L153 – 158) to clarify that we consulted other nudge-related reviews to support our objectives, however, no valuable information was retrieved. Therefore, we have added this section to inform our readers that this action was undertaken, but for the purposes of keeping a focused paper, additional information is omitted.

L153 – 158: “To date, systematic reviews of nudge interventions designed to improve individual health behaviors have focused on primary preventive behaviors for non-communicable disease control, such as healthy food choices, reducing alcohol consumption, smoking cessation, increasing physical activity, and self-management of chronic diseases [24-30]. To our knowledge, studies focusing on nudge strategies aimed at infectious disease control, and more specifically prevention and control of NTDs, have not been synthesized and this gap presents an opportunity for a scoping review.”

**Methods**

This part of the document is well written, and I don't have anything to add. I thought the authors were accurate in the adherence to the PRISMA-SR framework, and it served them well.

We thank the reviewer for the positive feedback on the methods section.

**Discussion**

I like that the authors put forward an ethical framework to aid in choosing nudge interventions. However, the section was difficult to follow along, and I encourage the authors to consider some kind of illustrative representation of their proposed framework. A tabular representation or some figure would be a practical addition to this manuscript. Furthermore, some kind of visual will be much easier to disseminate.

For example, you can have a table with three columns, High, Moderate, and Low. Each row is a criterion in the ethical framework, and relevant definitions are in each cell.

By providing the subheading “proposed ethical framework” in the discussion section, we gave the impression that we put forward a novel framework for choosing nudge interventions. Unfortunately, this is not the case, this section merely describes our results from the ethical assessment based on Engelen’s framework, which is structured as proposed by the reviewer in Table 3. The definitions are to be found in the column “labels”, including the assessments (H, M and L) in the last column “codes”. Figure 4 is a representation of the assessment/framework in action, it provides a visual overview of the ethical standards of this particular field, being the implementation of nudge strategies for NTDs.

We have included the following text in the discussion section for more clarity: L610 – 611: “…by using a modified categorization from Engelen’s criteria (Table 3). The findings show the majority of the included nudges were of moderate to high ethical standard (Fig 4).”

Given that the manuscript began with an argument about policy and agenda setting regarding NTDs, I think the discussion could benefit from a call back to the introduction. How might the findings contribute to further narrowing the policy and health agenda environments, either in specific countries, regions or globally?

We agree with the reviewer and have included in the first discussion paragraph (L543 – 556):

“Nevertheless, these studies also provide evidence and support for incorporate nudges in an integrated approach for combatting NTDs, as put forward by the NNN in their BEST framework [7]. Nudges have shown to be low-cost, and context-specific strategies that are relatively easy to include in complex interventions. The findings of this review provide an overview of an increasingly commonly used approach to promoting behavior change through altering people’s environment and behavior, by which we aim to facilitate future research in the field. Moreover, although horizontal health promotion approaches have become common practice, the results of this review point to the need for additional research that focuses on nudges in relation to specific health-promoting behaviors for combatting NTDs in particular. Several NTDs are acquired in specific contexts, and avoided by certain behaviors, that are not considered by horizontal health promotion practices: for instance, trachoma is prevented by face washing, schistosomiasis by avoiding contact with contaminated water, snakebite by wearing protective footwear. By altering a specific environment, nudges present an opportunity to focus on such context- and behavior-specific traits of NTDs. Therefore, future efforts should, in addition, take this specific nature of certain NTDs into account.”

**Figures**

Add some figure legends and labels to the figures you have at the end of your manuscript to explain what they mean. Typically, figures should serve as stand-alone from the text in that your reader should understand what they mean with minimal references to the text.

The figure legends were included in the written manuscript, following the paragraph where these are referred to. We were not able to upload these legends with our figure due to the submission process, we apologize for this inconvenience.

The systematic flow diagram is a bit confusing. A flow diagram should not have many arrows and boxes intersecting. Additionally, boxes in one category should not bleed over into others. For instance, in the Eligibility band, you have a box for Records Excluded after the full-text review (n=35). This 'Records Excluded' box extends into the 'Inclusion' band. Ideally, the only information in that band should be the' Studies included (n=33)' box.

We appreciate the feedback and the diagram had been adjusted for clarity as suggested by the reviewer

The flow diagram should also avoid using arrows with hatched lines that intersect with arrows with solid lines. This had been adjusted as suggested by the reviewer

For example, what are the arrows extending from the 'Records excluded (n=135)' back to the Full-text articles remaining box supposed to mean? If there are multiple reviews of the excluded text, then build those into the hierarchy vertically and lengthen the eligibility band.

We agree that the PRISMA diagram is somewhat confusing, but have tried to follow a similar example from Weston et al., 2018. The reason for this representation is that our process was more iterative than anticipated due to the vagueness of the term “nudge”. We have clarified the dashed lines in the text (L308 – 309) and figure legend:

L308 – 309: “Subsequently, the eligibility criteria were modified, uncertain records were examined for inclusion, and previously excluded records based on ‘ineligible intervention’ were re-assessed.”

Figure legend: [Fig 1 Flow diagram based on the Preferred Reporting Items for Systematic reviews and Meta-Analyses extension for Scoping Reviews (PRISMA-ScR) checklist (Tricco et al., 2018). Note that the selection process was iterative and resulted in reconsideration and subsequent inclusion of studies for eligibility assessment (dashed lines)]

We experimented with building the multiple reviews of the excluded text into the hierarchy vertically as suggested by the reviewer, but this extends the flow diagram over two pages, which we deem too long.

Check the below website for a PRISMA flow chart generator. I have found it helpful in the past. While this is a scoping review, the PRISMA flowchart typically used in systematic reviews can be effectively modified to suit this study's needs. <http://prisma.thetacollaborative.ca/>

We are thankful to the reviewer for sharing this link, but it doesn’t allow us to introduce multiple reviews of excluded text. We are keen on using this tool for our next, more linear review.

**REVIEWER 2**

**Reviewer 's Responses to Questions**

**Methods**

The methods are sound and compatible with the objectives to synthesis the existing body of evidence around nudging strategies within NTDs. The description of each step of the scoping review is well formulated. No statistical analysis was performed. Piloting to ensure validity of each tool is well documented, as well the process in which the ethical framework was adjusted.

- Do teh authors make efforts to contact the organizations/actors in the grey literature?

- However I would make a remark here that despite…

We did not make any attempt to contact authors and have clarified this in the limitation section as suggested by the reviewer (L690 – 693): “We did not make any effort to contact authors/organizations/actors with an incomplete description of the intervention in their study, since this would have increased the scope of the review and resulted in a significant delay of our work. We excluded all incomplete interventions and studies based on their reporting, and consequently may have missed additional relevant information.”

**Results**

There is room for improvement in terms of visualization of the results. Figure 1 is of low quality, while figure 2 and 3 provides only basic, if not fragmented, simplification of the included nudges and then the categories based on choice architecture.

We appreciate the feedback and have updated the figures and improved the quality, as well as the information (labels, etc.) presented in the figures. However, we did not change the content of the figures, and although we agree with the reviewer that these are rather simple visualizations of our results, it is an accurate representation of our data extraction process. Fig 4 is somewhat more complex since this represents an actual assessment of the data.

I appreciate the transparency in reporting the eligibility decisions taken by the review authors, including the reason of exclusion of certain study related to the understanding of 'nudge' strategies as part of an intervention. There is a mistake in Reference 32 in which the sentence below referred to Vietnam while the cited study is from Indonesia.

We thank the reviewer for his positive feedback on the eligibility decision section and have corrected this mistake.

There is little to be retained while reading the details on the study characteristics, especially in the 'intervention strategies', please make clear what constitutes complex versus moderate used by the authors.

As suggested by the reviewer, we have included this information in the results section for more clarity (L408 – 411), and it can also be found in the methods section under “data extraction” (L285 – 286).

L285 – 286: “i.e. largest number of intervention components implemented to a group participants; simple: 1-2, moderate: 3-4, and complex: >4”

In a similar vein, as considerable part of the included studies are actually RCT, it would be informative to include information on study design and as well (at least) outcomes of the studies included. It is somewhat moot to read about what strategies are being deployed but with little to no information on the performance/impact of such strategies.

We appreciate the opportunity to clarify and strengthen the discussion and conclusion sections regarding the need for more rigorous study designs in future studies. We have added a sub-section entitled “Study design” in the results section as proposed by the reviewer (L413 – 424). However, this only contains information on the research design of the included studies, and whether the design allowed for isolating and measuring the impact of separate behavior change strategies. Since this was generally not the case, we cannot provide any further information on the performance/impact of such strategies as requested by the reviewer. This knowledge gap is discussed in the section “Identifying areas for future research” (L583 – 603).

For the ethical assessment, I find that there should be an improvement in reporting the outcome, is the purpose of the team to show how to apply the said framework? Currently Figire 4 gives little information in conjunction with the other sections - i would like to know which studies/which nudge strategies correspond to which ethical standard (H, M or L).

This was previously included in S3 Appendix, which gave a structured overview of how the framework was applied and which studies/nudges corresponded to which ethical standard/code. We agree that this is substantial information to comprehend how the assessment was performed and how Fig 4 came about. Therefore, we include Table 4 in the manuscript, as suggested by the reviewer.

**Conclusions**

The conclusions presented by the authors are summarized in two - which I think quite commensurate with the data, but I think the 'robust study designs' mentioned should be elaborated further in relation with the current states found in the review.

We agree with the reviewer and have included the following text in the conclusion section (L725 – 728): “To date the field has primarily focused on whether or not an intervention achieves positive effects of the whole intervention package, without isolating and considering the effects of each behavior change strategy. This knowledge is necessary for achieving more targeted, and thereby more efficient and effective nudge strategies, or behavior change strategies in general.”

**Summary and General Comments**

Thank you to the editor and the authors for the chance to review this interesting manuscript. It is well written and overall following the standard of scoping review conduct very satisfactorily. However, I lack a bit the NTD explanation in the introduction, as well why particularly NTDs are targeted while overall most nudge strategies/studies are focusing on general health, if not child and environmental health. I have mentioned above that if the objective is to map what literature exists on the nudge strategies for NTDs, the discussions and conclusions do not go into the specificity of the findings to NTD, or in other words, in the end, this applies beyond NTD as well.

We have clarified both in the discussion and conclusion why NTDs are targeted and elaborated on findings specific for NTDs. We believe the explanation as to why we have mainly found studies focused on general health to fit the discussion section better, since it contributes to the interpretation and understanding of our findings, rather than elaborating on the results of our review at the introduction of our manuscript. Below are the sections included in the manuscript to address the reviewer’s comment:

Discussion (L543 – 556):

“Nevertheless, these studies also provide evidence and support for incorporate nudges in an integrated approach for combatting NTDs, as put forward by the NNN in their BEST framework [7]. Nudges have shown to be low-cost, and context-specific strategies that are relatively easy to include in complex interventions. The findings of this review provide an overview of an increasingly commonly used approach to promoting behavior change through altering people’s environment and behavior, by which we aim to encourage future research in the field. Moreover, although horizontal health promotion approaches have become common practice, the results of this review point to the need for additional research that focuses on nudges in relation to specific health-promoting behaviors for combatting NTDs in particular. Several NTDs are acquired in specific contexts, and avoided by certain behaviors, that are not considered by horizontal health promotion practices: for instance, trachoma is prevented by face washing, schistosomiasis by avoiding contact with contaminated water, snakebite by wearing protective footwear. By altering a specific environment, nudges present an opportunity to focus on such context- and behavior-specific traits of NTDs. Therefore, future efforts should, in addition, take this specific nature of certain NTDs into account.”

Conclusion (L718 – 723):

“Firstly, integrated health promotion approaches are currently dominating the field of behavioral NTD prevention and control efforts. Although effective for targeting a group of diseases at once, certain NTDs do not benefit from such an approach due to their specific nature and context. By altering a specific environment, nudges present an opportunity to focus on context-specific traits of NTDs. Future efforts should include such traits and investigate the different opportunities that nudges present.”

And also on the ethical framework and assessment, I would like to see the authors comment on the aspect aligned with decolonialization of global health, especially if the lack of autonomy, transparency etc indeed stemmed from asymmetrical power relation.

We thank the reviewer for pointing us back to the critique that nudges are paternalistic (i.e., lack autonomy, transparency, etc), and to the asymmetrical power relation that can be embedded within the countries, through the co-creation of knowledge with participants. We would like to clarify that our results did not point towards asymmetrical power relations stemming from systems of dominance between countries, since the nudges scored rather high on the “trust relationship” criteria. We have therefore included L 655 – 660, that elaborates on boosting as an alternative to nudging, more in line with the aspect of decolonization of global health, from a research vs. people perspective.

L655 – 664: “Boosting has been positioned by some in the field as being more in line with approaches that empower, given that they tend to be developed in a more participatory paradigm and are bottom up rather than top down. Approaches that aim to destabilize asymmetrical power relations through the co-creation of knowledge and strategies to improve health based on the lived experience of the participants themselves, are increasingly advocated [88]. Future research both with regards to boosting and nudging might benefit from greater attention to developing behavior change strategies in partnership with the target group, within a participatory approach whereby interventions are developed *with* rather than *on* populations. Such an approach would ensure interventions and strategies are firmly grounded in principles of transparency and democracy, thus reaching a higher ethical standard.”
