# Supplementary material for: Nudge strategies for behavior-based prevention and control of neglected tropical diseases: A scoping review and ethical assessment
Source: PLoS Negl Trop Dis. 2021 Nov 1;15(11):e0009239. doi: 10.1371/journal.pntd.0009239 (PMC8584752; doi:10.1371/journal.pntd.0009239)
Supplement: Attachment — Submitted filename: Cover letter_responses2.docx [file pntd.0009239.s005.docx]

**Cover letter - responses to the reviewer comments to submission PNTD-D-21-00223**

**Title:** Nudge strategies for behavior-based prevention and control of neglected tropical diseases: a scoping review and ethical assessment

Dear Editors,

We are again indebted and would like to extend our gratitude to the editors and reviewers for the thorough consideration and minor comment on our manuscript. Based the comment provided by reviewer 1, we have adjusted the figures to meet the journal’s requirements. To do so we have used the PACE tool as suggested, the figures now measure 7.5in W x 4.22in H and the fonts have been changed.

If by this adjustment the manuscript should be accepted for publication, we look forward to receiving the subsequent steps.

Please do not hesitate to get in touch if you have any further questions.

Yours sincerely,

Fiona Vande Velde

Postdoctoral fellow,

Dept. of Public Health Sciences,

Norwegian University of Life Sciences
